# Effects of Age and Parity on Mammary Gland Lesions and Progenitor Cells in the FVB/N-RC Mice

**DOI:** 10.1371/journal.pone.0043624

**Published:** 2012-08-30

**Authors:** Ahmed Raafat, Luigi Strizzi, Karim Lashin, Erika Ginsburg, David McCurdy, David Salomon, Gilbert H. Smith, Daniel Medina, Robert Callahan

**Affiliations:** 1 Cell and Cancer Biology Branch, National Cancer Institute, National Institutes of Health, Bethesda, Maryland, United States of America; 2 Children's Memorial Research Center, Robert H. Lurie Comprehensive Cancer Center, Northwestern University Feinberg School of Medicine, Chicago, Illinois, United States of America; 3 Office of the Director, National Cancer Institute, National Institutes of Health, Bethesda, Maryland, United States of America; 4 Laboratory of Cancer Prevention, National Cancer Institute; National Institutes of Health, Bethesda, Maryland, United States of America; 5 Department of Molecular and Cellular Biology, Baylor College of Medicine, Houston, Texas, United States of America; University of Edinburgh, United Kingdom

## Abstract

The FVB/N mouse strain is extensively used in the development of animal models for breast cancer research. Recently it has been reported that the aging FVB/N mice develop spontaneous mammary lesions and tumors accompanied with abnormalities in the pituitary glands. These observations have a great impact on the mouse models of human breast cancer. We have developed a population of inbred FVB/N mice (designated FVB/N-RC) that have been genetically isolated for 20 years. To study the effects of age and parity on abnormalities of the mammary glands of FVB/N-RC mice, twenty-five nulliparous and multiparous (3–4 pregnancies) females were euthanized at 16–22 months of age. Examination of the mammary glands did not reveal macroscopic evidence of mammary gland tumors in either aged-nulliparous or multiparous FVB/N-RC mice (0/25). However, histological analysis of the mammary glands showed rare focal nodules of squamous changes in 2 of the aged multiparous mice. Mammary gland hyperplasia was detected in 8% and 71% of the aged-nulliparous and aged-multiparous mice, respectively. Epithelial contents and serum levels of triiodothyronine were significantly higher in the experimental groups than the 14-wk-old control mice. Immuno-histochemical staining of the pituitary gland pars distalis showed no difference in prolactin staining between the control and the aged mice. Tissue transplant and dilution studies showed no effect of age and/or parity on the ability of putative progenitor cells present among the injected mammary cells to repopulate a cleared fat pad and develop a full mammary gland outgrowth. This FVB/N-RC mouse substrain is suitable to develop mouse models for breast cancer.

## Introduction

Advances in breast cancer treatment require better understanding of the genetics and biology of the disease. This requires the development of better *in vitro* and *in vivo* models of human breast cancer. Transgenic/knock-in and knockout mouse models have been used widely to observe *in vivo* the role of genes in initiation and progression of the disease. Their use has dramatically increased during the past decade. The ideal animal mammary gland tumor model should replicate the different stages of development of human breast cancer, such as pathogenesis, growth and progression with a 100% incidence in animal models and no occurrence in the control animals- at least during the defined period of experimental observation [1].

The FVB strain of mice is widely used for the generation of models of human breast cancer. Advantages in the use of this specific strain include vigorous reproductive performance and consistently large litters [2]. FVB mice also have large and prominent pronuclei in fertilized zygotes, which facilitate microinjection of DNA, and exhibit good survival of embryos following injection. The mouse mammary gland has been widely studied *in vivo* for elucidating the roles of hormones and growth factors in normal mammary gland development and function. Also, the mouse mammary gland is similar to the human breast in many aspects of hormonal regulation of cell proliferation [3]. Recently human embryonal carcinoma cells have been reprogrammed to human mammary cells in the mouse *in vivo* [4].

Wild-type aged-FVB female mice spontaneously acquire a series of abnormalities in several organs, such as lung, ovary and pituitary [5]. To be able to dissect and understand the abnormalities observed in the mammary glands of mouse models, it is important to know the phenotype of the normal mouse mammary gland used as control. Normal mammary development is affected by factors such as age, parity and substrain. Recent reports showed that mammary glands of aged-nulliparous FVB/NCr had the appearance of glands from mid-pregnant animals [6]. Also, these glands had lobulo-alveolar hyperplasia, with direct relationship between the percent of hyperplasia positive glands and age. About 75% of the mammary glands of aged FVB/NCr mice with hyperplasia had foci of squamous metaplasia [7]. In addition, mammary abnormalities were accompanied by proliferative lesions in the pituitary [6], [7]. Prolactin-secreting pituitary proliferations play a significant role in mouse mammary tumorigenesis generally by producing adeno-squamous carcinomas [7], leading to misinterpretation of phenotypes observed in animal models of mammary development and tumorigenesis [6], [7]. Therefore, we decided to investigate the role of age and/or parity on the potential development of abnormalities in the mammary and pituitary glands, uterus and ovaries, as well as the effects of age and/or parity on the ability of mammary stem cells to repopulate a mammary gland cleared fat pad (CFP).

The FVB mice used in this study are housed in our own colony and have been inbred and genetically isolated for the past 20 years. They therefore represent a substrain of the FVB/N mice (designated FVB/N-RC). The aged-multiparous (3 to 4 pregnancies) and the aged-nulliparous female mice were euthanized at 16–22 months of age. As control we used 14-week-old nulliparous female mice from the same colony. Neither age and/or parity resulted in spontaneous mammary tumor development or affected the ability of the mammary stem cells to repopulate the mammary gland; therefore we propose the FVB/N-RC mouse substrain as a suitable model for the development of mammary gland tumor models. This substrain will be made available to investigators interested in developing new models for breast cancer.

## Results

### Mammary morphology and histology

**Figure 1:**
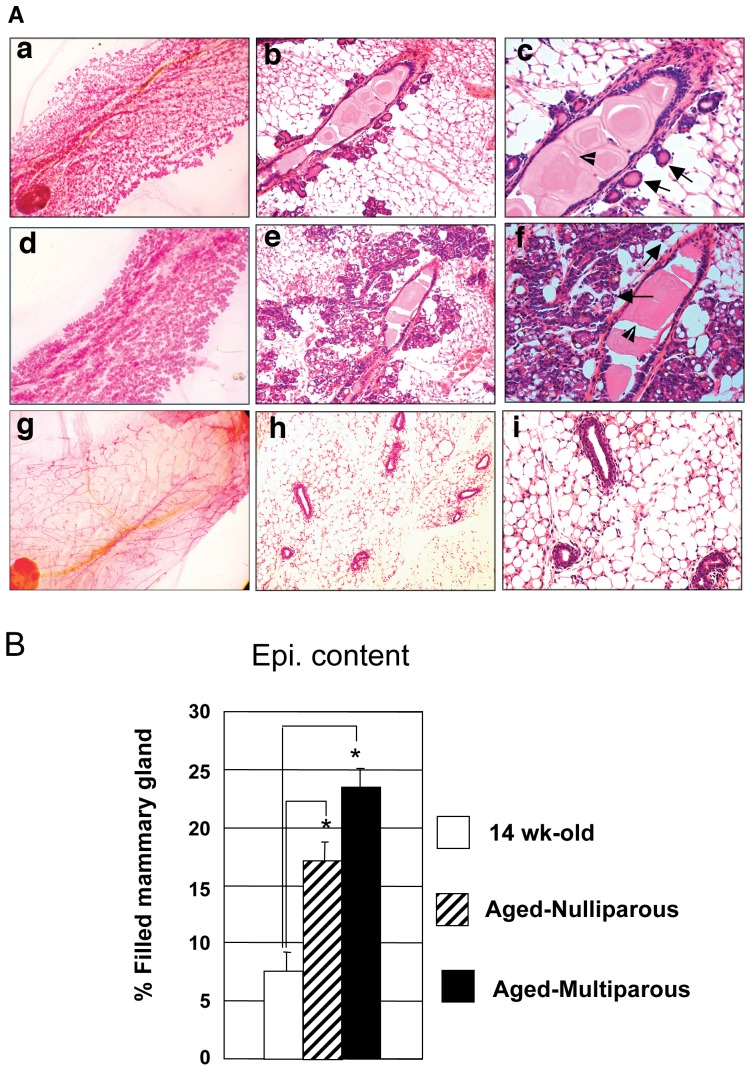
Analysis of morphology, histology and epithelial content of control, aged -nulliparous and aged-multiparous FVB/N-RC mammary glands. (**A**) Photomicrographs of aged-nulliparous (panel a, b and c), aged-multiparous (panel d, e and f) and 14-wk-old (panels g, h and i) control mice whole-mounts and histological secions. Whole-mounts of the aged-nulliparous (panel a compare to panel g) and aged-multiparous (panels d compare to panel g) mice had the appearance of mammary glands from pregnant mice. Similary sections of mammary glands of the aged-nulliparous (panel b and c) and aged-multiparous (panel e and f) mice had the appearance of those from pregnant mice. Mammary glands of the aged-nulliparous (Panel b and c) mice displayed few budding alveoli (arrows, panel c) emanating from primary ducts; the aged-multiparous glands consisted of higher order of branching containing extensive numbers of fully developed alveoli (arrows, panel e and f). Also, the ducts of the aged-nulliparous and multiparous mice are dilated and filled with secretory material (arrow head in panel c and f). The control 14-wk-old nulliparous female mammary glands were composed of primary ducts (Panel g, h and i). Magnification, Panels a, d and g are at 5×, panels b, e and h are at 10× and panels c, f and i are at 20×. (**B**) Quantification of epithelial content of mammary glands from 14-wk-old control, aged-nulliparous and aged-multiparous mice, was conducted on the whole-mounts using AxioVision 4 Module AutoMeasure Program. Aged-multiparous mice had the highest epithelial contents followed by the aged-nulliparous and the control mice. Data represent mean±SEM from ten mice per group. In Figure 1B * indicates a statistically significant difference of *p*<0.05.

Mammary glands of aged-nulliparous/multiparous females showed morphologic and histologic features of an early pregnant mouse mammary gland. Analysis of mammary morphology and histology showed high levels of budding alveoli in both the aged-nulliparous (Fig. 1A, panels a, b and c) and aged-multiparous (Fig. 1A, panels d, e and f) females compared to the 14-wk-old control mice (Fig. 1A, panels g, h and i) indicating a significant effect of age and/or parity. While 35% of the aged-nulliparous mammary glands showed few budding alveoli originating from the primary duct (Fig. 1A, panels b and c), 93% of the aged- multiparous females showed higher levels of alveolar content (Fig. 1A, panels e and f). The mammary glands of the control females showed no alveolar development (Fig. 1A, panels h and i). Only mammary glands of aged-nulliparous (Fig. 1A, panels b and c) and aged-multiparous (Fig. 1A, panels e and f) mice had dilated ducts filled with secretory material. Mammary glands of the nulliparous 14-wk-old control female mice were composed of primary ducts (Fig. 1A, panel g, h and i).

**Figure 2:**
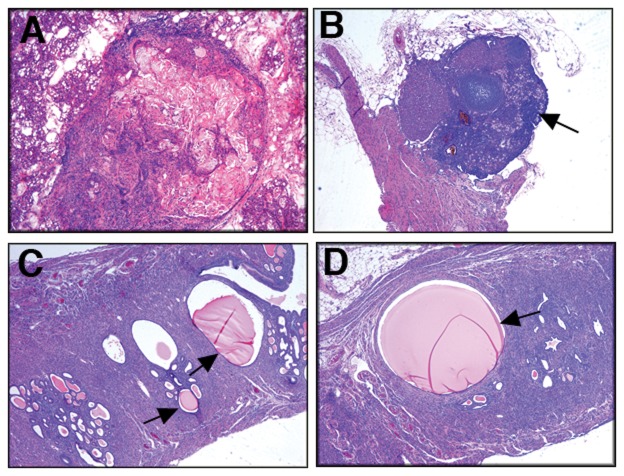
Mammary gland, ovary, pituitary and uterine anomalies. Representative H&E stained sections of mammary gland (A), ovary (B), and uterus (C & D). Focal nodules of squamous metaplasia were rarely observed in mammary sections of aged-multiparous mice (A), 10× magnification. Ovary anomalies occur frequently in the aged-multiparous mice, ovarian atrophy (panel B, arrow) was common in aged-multiparous mice, 5× magnification. Large fluid containing cysts suggestive of hydrometra (panel C arrow) and multiple smaller cysts were common in the uterus of aged-nulliparous females (panel D arrow), 5× magnification.

Epithelial content analysis showed effects for age, parity and both age and parity on the mammary gland epithelial contents (Figure 1B). The area of the mammary gland filled with the epithelial cells in aged-nulliparous mice was 2.4 times higher than the epithelial content of the control mice (17 vs. 7% respectively, Fig. 1B), indicating the effects of age. Epithelial contents in the mammary glands of the aged-multiparous mice was 3 times higher than the epithelial content of the control mice, (23 vs. 7% respectively; Fig. 1B), indicating the effects of both age and parity. While, the epithelial contents in the mammary glands of the aged-multiparous was 1.3 times higher than the epithelial contents of the aged-nulliparous gland (23 vs 17% respectively, Fig. 1B), indicating the effects of parity. This data, in addition to the histological analysis, suggests that the mammary glands of the aged-multiparous females did not fully regress after weaning of pups, resulting in side-branches and alveolar remnants. A similar observation for the FVB/N Lawrence Berkeley National Laboratory colony (LBNL) mouse subline has been reported by Nieto et al. [8]. Age and parity showed higher effect on the mammary hyperplasia than the effect of age only, as we compared the mammary glands of aged-multiparous mice versus the aged-nulliparous mammary glands. Sub-clinical hyperplasia was observed in 40% (10/25) and 8% (2/25) of the aged-multiparous and aged-nulliparous mice, respectively. Also, the average number of hyperplastic alveolar nodules/mammary gland was higher in the mammary glands of aged-multiparous than in the aged-nulliparous mice (2±0.75 Vs. 0.87±1.03 respectively). No hyperplasia was observed in the mammary glands of the 14-wk-old control group. Neither aged-nulliparous (0 out of 25) nor aged-multiparous (0 out of 25) mice showed macroscopic evidence of mammary gland tumors by 16 to 20 month of age. Only 2 of the aged-multiparous mice showed inflammatory infiltrates around rare foci of squamous nodules on histological examination (Fig. 2A).

### Ovary, uterus and pituitary morphology

Ovarian hormones control normal mammary gland development; in addition ovarian hormones play a critical role in tumor growth and development [9]. Histological examination of the ovaries revealed significant decrease in the number of follicles and corpora lutea, increased deposits of pigment and relative increase in interstitial tissue; in 10% of both aged-nulliparous and aged-multiparous mice, characteristics suggestive of ovarian atrophy (Fig. 2B). These results led us to investigate the morphology of the uterus, since the ovarian function is required for growth and function of the uterus [10]. For the uterus, 20% of the aged-nulliparous and aged-multiparous mice showed cysts of variable dimensions. Some cysts were extremely large and showed accumulation of material reminiscent of hydrometra (Fig. 2 C and D).

**Figure 3:**
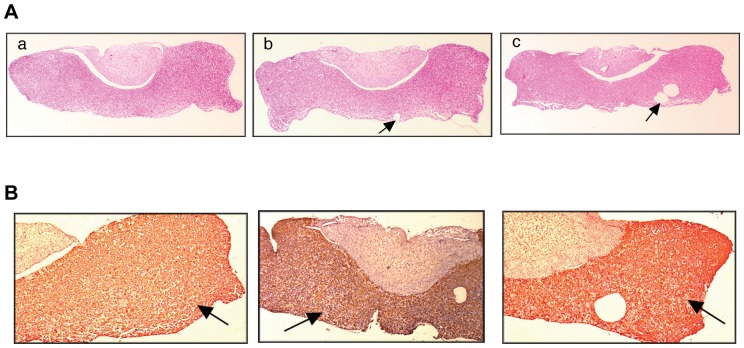
Pars distalis abnormalities and immunostaining. (**A**) Histological features of the pars distalis. H&E stained sections of pars distalis were examined in the 14-wk-old control (a), aged-nulliparous (b) and aged-multiparous (c) mice. Note the presence of cysts in the aged- nulliuparous (panel b) and aged-multiparous (panel c) females (arrow). Cysts were undetectable in the control 14-weeks-old (panel a) mice. (**B**) prolactin Immuno-histochemistry analysis of the pars distalis of 14-wk-old (a), aged-nulliparous (b) and aged-multiparous (c) mice showed uniform positive staining for prolactin in the control and experimental mice. Stained images illustrate the location of prolactin positive cells (arrows). Magnification 5×.

It has been reported previously that the abnormalities in the mammary glands of the aged-nulliparous and aged-multiparous mice are associated with lesions in the pars distalis and hyperplasia of the pituitary glands, resulting in high prolactin levels in the serum [6]. Therefore we investigated the morphology of the pars distalis of the pituitary. None of the experimental mice have had adenomas (Fig. 3A panels a, b and c) whereas cysts were observed in 50% of the aged-multiparous and aged-nulliparous pars distalis (Fig. 3A panel b and c). Immunohistochemistry analysis showed no altered staining of prolactin in the control (Fig. 3B, panel a) or experimental groups (Fig. 3B, panels b, and c).

### Serum hormones levels

Endocrine hormones interplay with the actions of several growth factors to influence mammary development [11]. Ductal elongation is directed by estrogen, growth hormone, insulin-like growth factor-I, and epidermal growth factor, whereas ductal branching and alveolar budding is influenced by additional factors such as progesterone, prolactin, and thyroid hormone [11]. Therefore, we investigated the effects of age and/or parity on serum levels of several key hormones. There was no significant difference between the control group and the experimental groups in the levels of circulating estrogen (E), thyroxin (T4) or growth hormone (GH). Triiodothyronine (T3) comparable levels in the aged-nulliparous and aged -multiparous mice serum indicates lack of parity effects on serum T3. While, the T3 levels are significantly higher in the serum of the aged-nulliparous and aged-multiparous females than the 14-wk-old control mice (97.41, 96.41 and 86.33 ng/DL respectively) (Table 1). Thus, this data suggests that the difference in serum T3 levels between the control and experimental mice is due to the age effect only. Bioactive prolactin analysis showed no significant difference among the three groups in the presence (50.1, 51.6 and 50.9 ng/ml) or absence (21, 19.93 and 21.1 ng/ml) of serum (Table 1).

**Table 1:** Effects of age and parity on serum hormone levels in the FVB/N-RC mice.

	Estrogen	T3	T4	GH	Bioactive Prolactin**	
Units	(pg/ml)	(ng/DL)	(μg/DL)	(ng/ml)	+serum	no serum
**Aged-nullip.**	47.70±6.11	97.41±1.18*	2.01±0.16	4.15±2.43	50.9±0.9	19.93±1.3
**Aged-multip.**	49.12±1.14	96.41±1.76*	1.96±0.15	3.92±0.38	51.6±0.5	21.10±0.7
**14-wk-old**	49.73±1.25	86.33±2.10	2.12±0.12	5.45±2.89	50.1±0.5	21.0±1.13

Serum was collected from 14-wk-old females, aged-nulliparous and aged-multiparous females and analyzed for the indicated hormones as described in Materials and Methods. No significant difference was observed in the serum levels of estrogen, thyroxin (T4), growth hormone (GH) or bioactive prolactin. Triiodothyronine (T3) was significantly higher in serum samples of the aged-nulliparous (*P<0.05) and aged-multiparous mice (*P<0.05 ) than the 14-wk-old control mice.

* =  P<0.05 vs. 14-wk-old.

** Serum levels of bioactive prolactin are measured in ng/ml.

### Proliferation, estrogen receptor alpha (ER-α) and progesterone receptor immunohistochemistry

**Figure 4:**
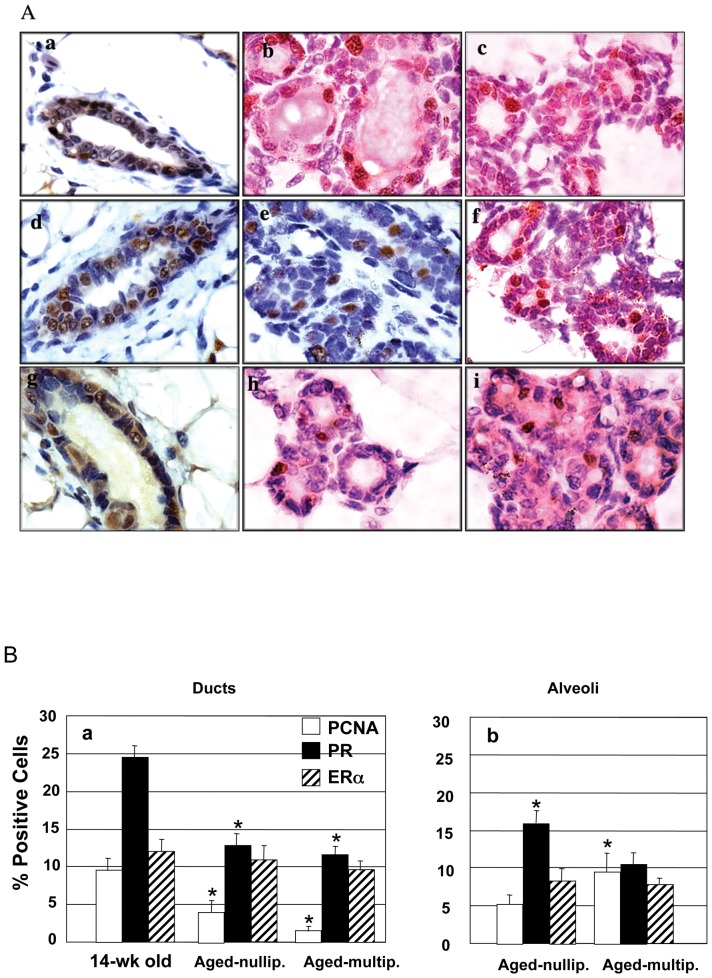
Effect of age and parity on proliferation, progesterone receptor and estrogen receptor-alpha (ER-α) levels in FVB/N-RC mice mammary glands. (**A**) Representative immuno-histochemistry sections of mammary tissue collected from 14-wk-old (panels a, d and g), aged-nulliparous (panels b, e and h) and aged-multiparous (panels c, f and i) female mice. Tissue was fixed and processed for age and parity associated proliferation marked by PCNA expression (Fig. 4A panels, a–c), progesterone receptor (Fig. 4A panels, d–f) and ER-α (Fig. 4A panels, g–i). Magnification 100×. (**B**) Proliferation, progesterone receptor and ER-α percent positive cells were scored and labeling index expressed as a percentage of positive nuclei of at least 3000 counted cells in mammary ducts (Fig. 4B panel a) and alveoli (Fig. 4B panel b). In the ducts, proliferation was significantly lower in the aged-nulliparous (*p*<0.05) and the aged-multiparous (*p*<0.05) mice than the 14-wk-old nulliparous mice. Also, proliferation was significantly lower (*p*<0.05) in the aged-multiparous than in the aged nulliparous mice mammary ducts. Progesterone receptor positive cells were significantly less in the ducts of the aged-nulliparous (*p*<0.05) and the aged-multiparous (*p*<0.05) mice than in the ducts of the 14-wk-old nulliparous mice. In the alveoli, proliferation was significantly higher in the aged-multiparous mice than in the aged-nulliparous (*p*<0.05). Also, progesterone receptor was more prevalent in the alveoli of the aged-nulliparous than in the aged-multiparous (*p*<0.05) mice mammary glands. In Figure 4B * indicates a statistically significant difference of *p*<0.05.

To further our understanding of the effects of age and/or parity on mammary morphology and to investigate the effects of these factors on epithelial cell density we looked at the epithelial proliferation (Fig. 4A, panels a, b and c). Labeling indices were determined in specific epithelial structures i.e., ducts (Fig. 4B panel a) and budding alveoli (Fig. 4B panel b). In the ducts, cell proliferation was significantly lower in the aged-nulliparous mice (p<0.05) and aged-multiparous mice (p<0.05) than in the 14-wk-old control (Fig. 4B panel a). Similarly, cell proliferation was significantly lower (*p*<0.05) in the aged-multiparous glands than the aged-nulliparous glands. This indicates a role for age and both age and parity, in mammary ductal epithelial proliferation. Conversely cell proliferation in alveoli was significantly higher (p<0.05) in the mammary glands of aged-multiparous than in aged-nulliparous mice (Fig. 4 B panel b).

The progesterone receptor (PR) knockout mouse demonstrated that progesterone is required for alveolar morphogenesis [12]. To determine whether the distinct mammary gland phenotypes observed in the aged-nulliparous/multiparous mice were due to differences in the levels of progesterone receptor, we compared the expression of PR in control and experimental mice using immuno-histochemical analysis (Fig. 4A, compare panels d, e, and f). In the ducts, the aged-nulliparous and aged-multiparous glands showed comparable levels of PR, indicating the lack of parity effect. However, PR levels are significantly lower in the ducts of the aged-nulliparous (p<0.05) and aged-multiparous (p<0.05) mice than in the 14-wk-old mice mammary ducts (Fig. 4B, panel a). This data indicates the effects of age on mammary gland PR levels. In the alveoli, PR levels were significantly higher (p<0.05) in the aged-nulliparous than the aged-multiparous mice (Fig. 4B, panel b), indicating an effect of parity on mammary gland PR levels. Hyperplastic structures observed in the aged-multiparous mammary glands were positive for PR and negative for PCNA (data not shown).

Epithelial PR is first detected at 7 weeks of age and this class of PR is regulated by estrogen [9]. Also treatment of ovarectomized mice with estrogen and progesterone resulted in side-branch formation and alveolar development [9]. Therefore we investigated the levels of estrogen receptor alpha (ER-α) in the ducts and the alveoli. Immuno-histochemical analysis of ER-α (Fig. 4A, panels g, h, and i) showed no difference in ER-α levels among the control and the aged-nulliparous and aged-multiparous mice (and Fig. 4B panel a and b).

### Transplant and dilution studies

**Figure 5:**
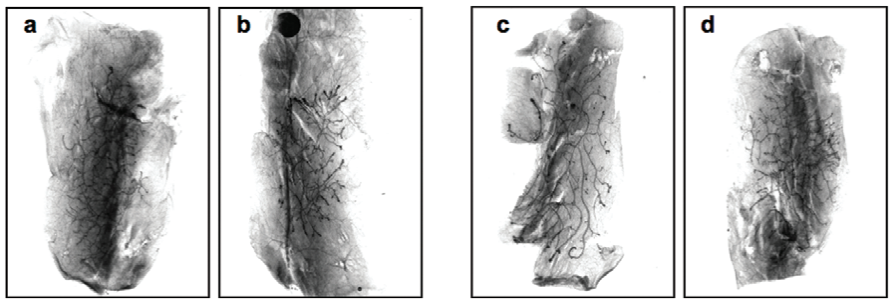
Transplantation results from implantation of dispersed, aged-nulliparous and aged-multiparous mammary epithelial cells into mammary fat pads of 3-wk old female mice. Representative whole-mounts of FVB/N-RC cleared fat pads transplanted with 250 (panel a and b) or 100 cells (panel c and d), collected from aged-nulliparous (panel a and c) and aged-multiparous (panel b and d), showing outgrowth filling of the fat pad. There was no difference in the results obtained with tissues from aged versus young donors regardless of parity.

**Figure 6:**
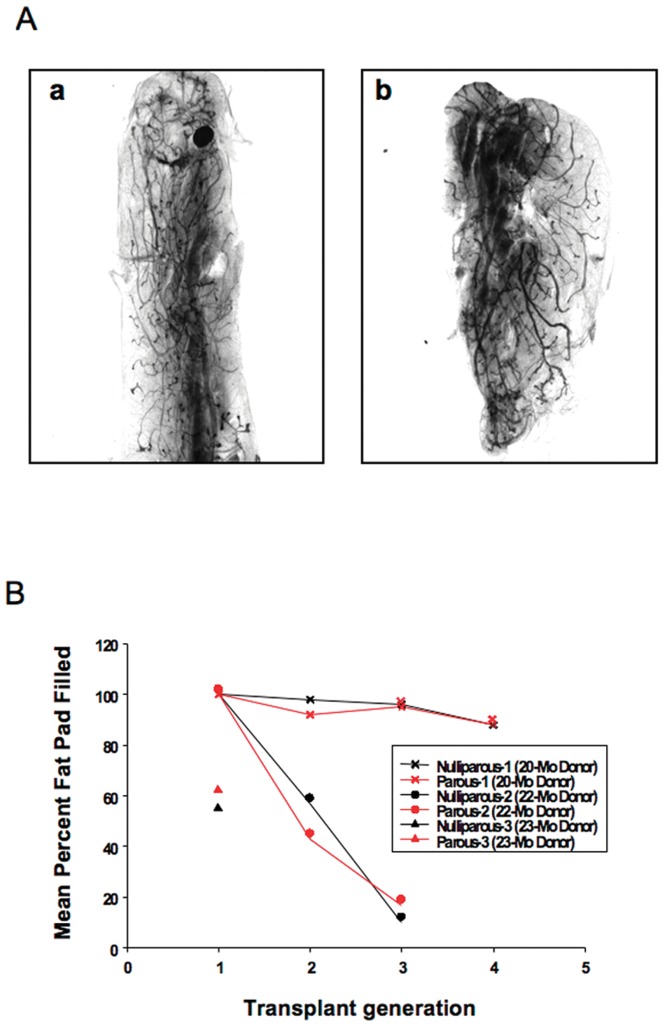
Transplantation results from implantation of mammary tissue fragments of, aged-nulliparous and aged-multiparous mammary epithelial cells into mammary fat pads of 3-wk old female mice. Hosts were maintained as virgins for 8 weeks after the transplant. At the end of the 8 weeks, mammary glands were collected for morphology analysis and fragments were collected for the serial transplantation. All fragments contributed to complete outgrowth of the mammary gland when implanted into cleared mammary fat pad. (**A**) Portrays a representative whole-mount image of the fifth generation transplant (panel a) of an outgrowth of aged-nulliparous mammary tissue fragment into the cleared mammary fat pad (CFP), (panel b) shows the comparable outgrowth of aged-multiparous mammary tissue fragment into the CFP. (**B**) Serial transplantation results from transplant of paired aged-nulliparous and aged-multiparous mammary donor fragments into cleared mammary fat pads. Tissue fragments were collected from 20-month old mice (20-Mo-Donor), 22 month old mice (22-Mo-Donor) or 23-month old mice (23-Mo-Donor) as donors for the first transplant generation. Each donor mouse represented an independent experiment. In all cases fragments from the matched donors produced comparable mammary outgrowths.

To examine the effects of age and/or parity on mammary stem cell (MaSC) transplantation potency, we conducted two assays, a serial dilution transplantation study and serial transplantation of mammary tissue fragments. Outgrowth took place in both assays, independent of the numbers or source of cells (Fig. 5 and Table 2) or source of fragments (Fig. 6A). In the cell dilution assay, mammary outgrowth took place at all dilutions (Fig. 5 and Table 2). There was no statistical difference in takes or amount of fat pat filled between aged and young donors. No hyperplastic lesions were observed in the mice transplanted with epithelial cells from either the aged-nulliparous or aged -multiparous female mice donors. Thus, neither age and/or parity had a significant effect on the MaSC activity. In the serial mammary fragment transplantation assay implants behaved quite differently but within the same individual sets there was no statistical difference in the amount of fat pad filled between the mammary fragments from the aged-nulliparous versus the aged-multiparous (Fig. 6 B). Therefore, transplanted mammary fragments containing MaSC from aged-nulliparous versus aged-multiparous are capable of self-renewal and proliferations over several transplant generations and persist in the transplant outgrowth populations until growth senescence in a similar fashion. In all cases the mean percent of outgrowth was at least 30% of the CFP or greater. These data show that age and/or parity does not affect the ability of MaSC in multiparous versus nulliparous transplants to repopulate the mammary fat pad.

**Table 2:** Number of positive outgrowths (takes) originating from the implant of mammary gland cells into cleared fat pads.

	5000 cells	1000 cells	500 cells	250 cells	100 Cells
**Aged-nulliparous**	10/11	7/11	7/12	6/13	6/16
**Aged-multiparous**	10/12	11/12	8/12	12/16	6/16
**14-wk-old**	ND	15/16	12/15	8/12	6/22

Mammary glands of FVB/N-RC female mice at 3 weeks of age were surgically cleared of indigenous mammary epithelial in both #4 fat pads and implanted with mammary fragments from 14-wk-old, aged-nulliparous or aged-multiparous glands. ND, not done.

## Discussion

Mouse mammary gland development is well studied and its response to hormones and growth factors is well understood. This makes it a good candidate to develop breast cancer models. Understanding breast caner initiation, promotion and progression involves an increasing number of experimental mouse models. The FVB/N mouse strain is commonly used in these models. The ideal mouse model should not develop mammary abnormalities in the control group-at least during the time period used for comparison with the experimental group. Recent reports of the development of spontaneous mammary hyperplasia/tumors in the FVB/N mice [6], [7] led us to investigate the incidences of mammary abnormalities in our own colony. The FVB/N-RC mice have been inbred for the last 20 years, separate from FVB/N mice from other sources and therefore represent a substrain of the FVB/N. Our results are in agreement with Nieto et al. results [8], they used a small cohort of multiparous FVB/N female mice from the LBNL. These mice did not have pituitary abnormalities or high serum prolactin levels even though some of these multiparous females have had mammary hyperplasia [8]. Thus genetically isolated substrains of FVB/N mice might develop separate mammary phenotypes. The difference in the phenotypes between the FVB/N-RC and the FVB/N are most probably due to a founder effect. Michael Potter [13] tested several sublines of BALB/c mice for their ability to develop plasmacytomas in response to mineral oil injections. Only BALB/c sublines from two different sources developed plasmacytomas and the rest were resistant. These results illustrate the effects of the genetic background of the founder and its effect on the phenotype. Therefore for valid breast cancer mice models, it is important to make the control group from the same subline used to make the transgenic experimental model.

The majority of mammary gland development takes place post-natally. These developmental events are driven by the ovarian hormones estrogen and progesterone. Where ductal elongation during puberty is driven mainly by estrogen and the alveolar formation is driven by estrogen and progesterone in addition to other growth factor, hormones and integrins [9]. No significant difference was observed in serum levels of estrogen among the three groups of mice. Estrogen down-regulates its own receptor [9], [14] and this is considered as an indication of the fact that the estrogen receptor is functional. Therefore the comparable serum estrogen levels in the control and the experimental mice, explain in part the similarity in the ER-α levels in the ducts and alvoeli of the three groups. It has been reported that low levels of ER-α expression are observed in women of low breast cancer risk [15], [16]. Also, overexpression/dergulation of ER-α in C57BL/6 mice resulted in ductal hyperplasia development, even in the absence of estrogen [16]. Therefore, the comparable ER-α levels in the control and experimental groups indicate equal risk of mammary tumor development among the control and experimental mice.

The high proliferation rate in the 14-wk-old control mice could be due in part to the low T3 levels in this group (Table 1). As a matter of fact the control group showed the lowest levels of T3 followed by the aged-nulliparous and the aged-multiparous groups. It has been shown that T3 can reduce the proliferation of mammary epithelial cells and it inhibits the expression of cyclin D1 [17]. In addition, other groups have suggested that the effects of thyroid hormones appear to depend on their ratio to prolactin, estrogen or progesterone [11]. The relationship between breast cancer and thyroid diseases is controversial [18]. In primiparous mice, hyperthyroid did not result in increases in the incidence of mammary tumors in comparison to control mice when prolactin levels were maintained at normal levels [19]. Therefore it is not feasible to predict that aged-nulliparous and/or the aged-multiparopus mice will develop tumors based solely on their high T3 serum hormones levels.

Involution takes place after lactation, where massive cell death takes place. This results in the remodeling of the mammary gland and disappearance of the side-branches. The high epithelial contents in the aged-nulliparous and aged-multiparous females indicate failure of mammary regression after lactation, resulting in the maintenance of the alveoli and side-branches. This is a typical pattern in the aged-multiparous females [8]. In the aged-nulliparous females the continuous exposure of the mammary glands to the estrus cycle hormones could explain the development of the alveoli, and the effect of age on mammary morphology.

Stem cells are located along the entire mammary tree and are present in the different developmental stages of the mammary gland [20]. Experiments performed in mice have demonstrated that any portion of the mammary tree at any developmental stage, or any age contain cells that are capable of repopulating the mammary stroma and undergo the complete developmental cycle of the parenchyma [20], [21]. These experiments convincingly demonstrate that totipotent stem cells exist throughout the mammary parenchyma tree and are not localized to just the terminal portions of the mammary tree. Our data demonstrate that mammary epithelial cells and their progeny were able to contribute extensively to ductal growth and elongation upon transplantation to epithelium-cleared mammary fat pads. In the limiting dilution transplantation study, cells also proliferated and produced ductal outgrowths, indicating that the multipotent mammary stem cells activity is not affected by parity and/or age.

In summary the dilution studies indicate that there is no evidence for a loss of regeneration potential of mammary cells as a result of age and/or pregnancy. These data support the original observations of Young et al. [22] and the recent results of Britt et al. [23] and do not support the results of Siwko et al. [24]. The present study is the most comprehensive study on the question of the relationship between parity and mammary stem cells (i.e., the ability to regenerate a mammary gland upon transplantation), the data are compelling for support of the overall conclusion. Thus, there is no evidence for a loss of regeneration potential of mammary cells as a result of age or parity. In conclusion each isolated mice substrain might develop a separate mammary phenotype. Therefore, in the mouse models studies, it is important to include age and parity matched controls from the same colony. Also, neither age nor parity affected the regeneration potential of MaSC.

## Materials and Methods

### Ethics Statement

All mice were housed in Association for Assessment and Accreditation of Laboratory Animal Care–accredited facilities in accordance with the NIH Guide for the Care and Use of Laboratory Animals. The National Cancer Institute (NCI) Animal Care and Use Committee approved all experimental procedures.

### Mice

All the FVB mice used in this study have been maintained by sibling mating in our colony for the last 20 years and therefore represent a substrain (designated FVB/N-RC) of the FVB/N mice. Twenty-five FVB/N-RC female mice were set up for breeding at the age of ten-weeks. After the third or forth pregnancy, the breeding pairs were separated and the females were housed until 16–20 months of age. Another 25 females were maintained nulliparous to 16–20 months of age. The control group was 14-week-old nulliparous mice. Tissue and blood samples were collected from all mice at diestrus. All mice were kept according to standard governmental and NIH regulations. Mice had access to water and food ad libitum during the experiment, fed 5L79 diet (Charles River, Wilmington, MA, USA) and lived in a 12 hour dark/light cycle.

### Tissue preparation and epithelial content analysis

Mice were euthanized at the indicated ages. Mammary glands, pars distalis, uterus and ovary tissues were fixed in freshly prepared 4% paraformaldehyde in phosphate-buffered saline (PBS) (pH 7.4) for 2 h, rinsed through several changes of buffer, dehydrated in ethanol and stained and examined grossly under a dissecting microscope, or embedded in paraffin. Paraffin sections (5 micron) were placed on slides, deparaffinized and stained with H&E. Mammary whole-mounts and histology sections were prepared from the fourth abdominal gland as described previously [25].

### Immuno-histochemistry

Immuno-histochemistry was carried out with the ABC method according to the manufacturer's protocol (Vector Laboratories Inc., Burlingame, CA, USA). Primary antibody incubation was carried out overnight at 4°C: ERα (SC-542, Santa Cruz, Santa Cruz, CA, USA), diluted 100X; progesterone receptor (Dako, California, USA), diluted 100X; prolactin (7770-2949, AbD Sero-Tec, MorphoSys, Oxford, UK), diluted 1000X. Primary antibodies were diluted in 1%PBS-BSA. The biotinylated anti-rabbit (PK-6101, Vector Laboratories Inc., Burlingame, CA, USA) secondary antibody was diluted according to the manufacturer's recommendations. Labeling index was determined in at least a total of 3000 cells in each experiment.

### Serum collection and analysis

Serum was analyzed from 3 independent pools of control, aged-nulliparous or aged-multiparous FVB/N-RC female mice. Each blood pool was derived from three animals, obtained by exsanguinations at 2 PM. Sera was collected and subsequently stored at −20°C until assayed. Serum estrogen, triiodothyronine, and thyroxin were assayed by RIA (Ani Lytics. Inc., Gaithersburg, MD); growth hormone was quantified by ELISA (Ani Lytics, Inc.). Serum prolactin (PRL) levels were determined using a previously described bioassay that utilizes the PRL-responsive Nb2 lymphoma cell line [26].

### Tissue transplant and dilution studies

To characterize the effects of age and parity on mammary gland progenitor cell population and their ability to repopulate the mammary cleared fat pad (CFP); 3 different sets of aged-nulliparous and aged-multiparous mice were used. We employed two assays. In the first assay, 5000, 1000, 500, 250 or 100 cells from 14-wk-old FVB/N-RC, 20–23 month aged-nulliparous or 20–23 month aged-multiparous mammary glands were injected into the CFP of 3-wk old female FVB/N-RC mice. In each recipient mouse, one side received the nulliparous cells and the contralateral side received the multiparous cells. The second assay is serial transplantation in which we transplanted 1 mm^3^ fragments of mammary duct from 14-wk-old, aged-nulliparous or aged-multiparous mammary mice into the cleared fat pads of 3-week-old FVB/N-RC female mice. Eight to ten weeks after the implantation, recipient mice were euthanized, and a mammary tissue fragment was collected and used for serial transplantation. The serial transplantation was carried over for five consecutive times. Whole-mounts were prepared as previously described [25].

### Statistics

Quantitative values are represented as the mean of at least 3 experiments. All *in vivo* experiments were repeated at least 3 times, and at least 5 mice were used in each experiment. The statistical significance of difference between groups was determined by the Wilcoxon rank sum test. Comparisons resulting in *p*-values less than 0.05 were considered statistically significant and are indicated in the figure with asterisk (*). The effects of age were tested by comparing the 14-week-old control group data versus the aged-nulliaprous group data. The effects of age and parity were investigated by comparing the data of the 14-week-old control group to the data of the aged-multiparous group. While the effects of parity were investigated by comparing the data of the aged-nulliparous group to the aged-multiparous group.
